# Lifestyle changes and patient-reported outcomes over five years in a sample of people with multiple sclerosis after a single multimodal intensive lifestyle education workshop

**DOI:** 10.1007/s10072-024-07811-2

**Published:** 2024-10-21

**Authors:** Jeanette Reece, George A Jelinek, Elasma Milanzi, Steve Simpson-Yap, Sandra L Neate, Keryn L Taylor, Pia L Jelinek, Rebekah Davenport, William Bevens, Maggie Yu

**Affiliations:** 1 Neuroepidemiology Unit, Melbourne School of Population and Global Health, The University of Melbourne, Parkville, VIC Australia; 2 Biostatistics Unit, Centre for Epidemiology and Biostatistics, Melbourne School of Population and Global Health, The University of Melbourne, Parkville, VIC Australia; 3 Florey Institute for Neuroscience and Mental Health, The University of Melbourne, Parkville, Australia; 4 Menzies Institute for Medical Research, University of Tasmania, Hobart, Australia; 5 Interface Psychiatry Private Practice, Wellington, New Zealand; 6 Woodlands Family Practice, Woodlands, WA Australia; 7 Melbourne School of Psychological Sciences, The University of Melbourne, Parkville, Australia; 8 Institute for Clinical and Translational Science, University of California, Irvine, US

## Abstract

**Introduction:**

Modifiable lifestyle risk factors for progression of multiple sclerosis (MS) have been increasingly studied. This study employed a single-group design involving a one-off intensive live-in educational workshop on lifestyle modification for people with MS. We aimed to examine changes in a range of clinical and lifestyle variables and quality of life, self-efficacy, physical impact of MS and disability from baseline to 3- and 5-years post-intervention.

**Methods:**

95 participants completed the baseline survey. Data included lifestyle risk factors of diet quality, meat and dairy consumption, omega 3 and vitamin D supplementation, physical activity, stress reducing activities, and smoking status, and use of disease-modifying therapies (DMTs). Patient-reported outcomes included health-related quality of life, self-efficacy, physical impact of MS and disability. Generalised estimating equation models were used to account for within-participant correlation over time.

**Results:**

Sixty participants (63.2%) provided data at 3- and 5-years. Significant improvements in diet quality, omega 3 supplementation, and non-smoking were seen at both timepoints. Use of DMTs and disability remained unchanged. Mental (8.8- and 6.9-point) and physical (10.5- and 7.3-point) quality of life, and self-efficacy (2.4- and 1.9-point) improved significantly at 3- and 5-years, respectively. Physical impact of MS reduced from baseline to 3-years (-3.7 points) with a trend towards reduction at 5-years (-2.9 points; *p* = 0.079).

**Conclusions:**

Education on lifestyle modification can lead to lifestyle modification and short and long-term improvements in mental and physical wellbeing outcomes. Results suggest potential value in lifestyle modification as an adjunctive component to standard therapy for MS.

## Introduction

Once considered an incurable, progressively disabling neurological illness, multiple sclerosis (MS) is now a treatable chronic condition with a range of widely prescribed and reasonably effective disease-modifying therapies (DMTs). In recent decades, modifiable lifestyle risk factors for progression of this chronic illness have been increasingly identified and studied. Despite a lack of large scale, well-designed randomised controlled trials verifying the potential benefit of these lifestyle factors, diet, physical activity, sun exposure, vitamin D intake, and stress reduction have been associated with health outcomes and disability progression, demonstrated in a range of cross-sectional and longitudinal observational studies [1–7]. Notably, findings from a large international study of people living with MS, the HOLISM (*H*ealth *O*utcomes and *L*ifestyle *I*n a *S*ample of people with *M*s) study, which examined these risk factors and their changes over 7.5 years’ follow-up, revealed associations between changes in risk factors and a spectrum of clinically relevant patient-reported outcomes, including disability, fatigue, depression, self-efficacy, and quality of life (QOL) [8–14]. Few multimodal lifestyle interventions have been developed for people living with MS [15] and long-term follow-up beyond 1-year is limited, preventing insights into the longevity of their effects.

In parallel with HOLISM, we have examined interventional data on a smaller cohort of people with MS attending intensive live-in educational workshops outlining why and how these risk factors can be modified. One and five year follow-up analyses of 165 participants in this STOP MS (*ST*udying *O*utcomes of *P*eople attending *MS* retreats) study showed continued improvements in health-related quality of life (HRQOL) [16]. More recently, our group reported sustained improvements in HRQOL and lifestyle modifications at 1-year and 3-year follow-ups among a subset of 95 of these workshop participants [17].

The current study aimed to examine the extent to which changes in lifestyle risk factors following this one-off educational workshop were maintained. Clearly, in order to inform future study design and choice of outcomes, it was important to document any accompanying changes in mental and physical HRQOL, disability, physical impact of MS, and self-efficacy, assessed at 3-and 5-years post-intervention. This can contribute to the growing body of evidence surrounding whether lifestyle modification can be used as an adjunctive, self-management component to standard therapy for MS.

## Materials and methods

### Lifestyle workshop

The workshop has previously been described in detail [16, 18, 19]. Briefly, people with MS responded to public advertisements for a five-day live-in educational workshop. Lead facilitators were specialist medical practitioners (GAJ and KLT), with lived experience of MS and with developing and applying the multimodal lifestyle program utilised in the workshop. Interactive presentations were made around the key risk factors and how to modify them, along with support from other trained facilitators around stress reduction and emotional wellbeing. All food and beverage provided was in line with program guidelines and there were experiential sessions around food preparation, physical activity, and stress reduction. The workshops ran two to three times annually beginning in 2002.

### Design and participants

Detailed data on demographic characteristics, lifestyle factors and patient-reported health outcomes were collected from 95 participants, all of whom had a physician-confirmed MS diagnosis and had attended a single educational workshop in 2012 or 2013, hosted at a dedicated retreat facility in rural Victoria, Australia. These participants completed a detailed baseline survey and were invited to participate in both the 3- and 5-year follow-up surveys. Three-year follow-up data have previously been reported from this group [17].

### Ethics

Approval for the study was granted by the Human Research Ethics Committee of the University of Melbourne (HREC ID: 0723028.1).

### Measures

#### Demographics and clinical factors

This study assessed a range of demographic, clinical and lifestyle factors, all previously described [17]. Age was calculated using the reported date of birth and dates of survey completion. We queried data on sex (male, female), highest level of education (no formal schooling to postgraduate degrees), employment status (full-time work, part-time work, self-employed, stay-at-home, retired due to age/medical reasons/disability, and unspecified), marital status (married, cohabiting/partnered, separated/divorced/widowed, single) and MS type (relapsing-remitting, benign, secondary progressive, primary progressive, progressive relapsing). Variables were re-categorised as indicated in Table 1.

**Table 1:** Participant characteristics between excluded sample and study sample at baseline and study sample over time

	Excluded participants *N* = 35	Study participants *N* = 60		
	Baseline	Baseline	3-year	5-year
**Demographics and clinical factors**				
	**N (%)**			
Sex				
Male	11 (34.4)	15 (26.3)	15 (26.3)	15 (26.3)
Female	21 (65.6)	42 (73.7)	42 (73.7)	42 (73.7)
Missing	3 (8.6)	3 (5.0)	3 (5.0)	3 (5.0)
University education				
No	13 (37.1)	13 (21.7)	13 (21.7)	13 (21.7)
Yes	22 (62.9)	47 (78.3)	47 (78.3)	47 (78.3)
Missing	0 (0.0)	0 (0.0)	0 (0.0)	0 (0.0)
Paid employment				
No	9 (25.7)	15 (25.4)	13 (22.4)	15 (26.3)
Yes	26 (74.3)	44 (74.6)	45 (77.6)	42 (73.7)
Missing	0 (0.0)	1 (1.7)	2 (3.3)	3 (5.0)
Partnered				
No	10 (28.6)	11 (18.3)	14 (24.1)	14 (24.6)
Yes	25 (71.4)	49 (81.7)	44 (75.9)	43 (75.4)
Missing	0 (0.0)	0 (0.0)	2 (3.3)	3 (5.0)
MS phenotype				
Benign	0 (0.0)	0 (0.0)	3 (5.1)	5 (8.6)
RRMS	28 (80.0)	41 (68.3)	43 (72.9)	38 (65.5)
PPMS/PRMS/SPMS	2 (5.7)	6 (10.0)	3 (5.1)	8 (13.3)
Unsure	5 (14.3)	13 (21.7)	10 (17.0)	7 (12.1)
Missing	0 (0.0)	0 (0.0)	1 (1.7)	2 (3.3)
	**Mean (SD)**			
Age	40.9 (9.9)	45.5 (10.5) *	48.6 (10.7)	50.5 (10.8)
PDDS	1.4 (1.9)	1.3 (1.8)	1.3 (1.8)	1.4 (2.0)
MSIS-PHY	33.0 (14.0)	34.8 (13.5)	33.1 (14.6)	33.2 (15.7)
Mental QoL	70.7 (19.0)	68.4 (18.7)	77.0 (16.6)	75.4 (17.2)
Physical QoL	67.1 (19.4)	65.2 (18.4)	75.3 (17.3)	70.7 (18.7)
PAM-13	52.7 (6.6)	54.1 (5.6)	56.8 (6.7)	56.7 (5.7)
**Healthy lifestyle factors**				
Diet quality (DHQ), Mean (SD)	86.5 (9.0)	84.3 (11.1)	87.7 (6.8)	85.6 (8.5)
No dairy or meat consumption				
No	13 (40.6)	28 (48.2)	12 (20.3)	17 (29.3)
Yes	19 (59.4)	30 (51.7)	47 (79.7)	41 (70.7)
Missing	3 (8.6)	2 (3.3)	1 (1.7)	2 (3.3)
Vitamin D ($\:\ge\:$5,000 IU/day)				
No	11 (39.3)	25 (50.0)	25 (48.1)	28 (52.8)
Yes	17 (60.7)	25 (50.0)	27 (51.9)	25 (47.2)
Missing	7 (20.0)	10 (16.7)	8 (13.3)	7 (11.7)
Sun exposure ($\:\ge\:$15 min/day; $\:\ge\:$3 day/week)				
No	22 (68.8)	35 (60.3)	36 (61.0)	32 (56.1)
Yes	10 (31.3)	23 (39.7)	23 (39.0)	25 (43.9)
Missing	3 (8.6)	2 (3.3)	1 (1.7)	3 (5.0)
Flaxseed oil supplementation				
No	18 (51.4)	43 (71.7)	16 (26.7)	15 (25.0)
Yes	17 (48.6)	17 (28.3)	44 (73.3)	45 (75.0)
Missing	0 (0.0)	0 (0.0)	0 (0.0)	0 (0.0)
Meditation (weekly)				
No	24 (75.0)	38 (65.5)	32 (54.2)	29 (51.8)
Yes	8 (25.0)	20 (34.5)	27 (45.8)	27 (48.2)
Missing	3 (8.6)	2 (3.3)	1 (1.7)	4 (6.7)
Physical activity				
Low	5 (15.6)	13 (24.1)	13 (22.4)	9 (16.8)
Moderate/high	27 (84.4)	41 (75.9)	45 (77.6)	45 (83.3)
Missing	3 (8.6)	6 (10.0)	2 (3.3)	6 (10.0)
Non-smoker				
No	15 (46.9)	23 (40.0)	1 (1.7)	1 (1.7)
Yes	17 (53.1)	35 (60.3)	57 (98.3)	57 (98.3)
Missing	3 (8.6)	2 (3.3)	2 (3.3)	2 (3.3)
Disease-modifying therapy use				
No	7 (33.3)	8 (18.2)	12 (21.8)	18 (32.1)
Yes	14 (66.7)	36 (81.8)	43 (78.2)	38 (67.9)
Missing	14 (40.0)	16 (26.7)	5 (8.3)	4 (6.7)

Note. Differences of variables by baseline health communication engagement were assessed using logistic regression. Percentages are based on complete data for each factor. Missing data percentages are based on the total cohort sample and are not included in the non-missing data percentage. *=*p* < 0.05 for differences between study participants and excluded participants (reference category). Abbreviations: MSIS-PHY = Physical impact of Multiple Sclerosis Impact Scale; PAM = Patient Activation Measure; PDDS = Patient-determined disease steps; PPMS = Primary progressive multiple sclerosis; PRMS = Progressive-relapsing multiple sclerosis; RRMS = Relapsing-remitting multiple sclerosis

#### Lifestyle factors

Diet was assessed using the modified Diet Habits Questionnaire (DHQ). From this, two binary variables were derived to indicate whether participants consumed dairy and meat, respectively (0 = do not consume; 1 = consume). Vitamin D intake was based on self-reported IU dose and frequency, from which the average daily dose was estimated. A threshold of 5,000 IU/day was set as a cut-off for analysis. Omega-3 supplementation with fish or flaxseed oil was queried and included in the analysis versus no supplementation. Physical activity was measured via the International Physical Activity Questionnaire-Short Form, which recalls the frequency and duration of walking, along with moderate and vigorous-intensity activities over a 7-day period. For analysis purposes, activity levels were categorized into low versus moderate/high. Smoking status was dichotomised as current versus never/ex-smoker. Adequate sun exposure was queried from frequency and duration of sun exposure and dichotomised as 1 = ≥ 15 min/day ≥ 3 days/week vs. 0 = less frequently. Meditation frequency and duration were assessed using two researcher-devised items, which were collapsed into a binary variable for analysis (1 = meditated at least weekly; 0 = less frequently) due to the limited number of participants meeting higher frequency recommendations.

#### Patient-reported outcomes

Disability was assessed using the Patient-Determined Disease Steps (PDDS) [20]. PDDS scores range from 0 (normal) to 8 (bed-bound) and have been cross-validated against the Expanded Disability Status Scale (EDSS) [21]. The physical impact of MS was assessed using the physical component of the Multiple Sclerosis Impact Scale (MSIS-PHY). MSIS-PHY is a 20-item subscale where higher scores indicate a greater degree of physical impairment due to MS. An 8-point change on this scale is considered clinically meaningful [22]. HRQOL was measured with the Multiple Sclerosis Quality of Life survey (MSQOL-54). Mental and physical health composite scores were reported, ranging from 0 to 100, with higher scores denoting greater HRQOL. A 5-point change in either of these composite scores has been regarded as clinically meaningful [23]. Self-efficacy was assessed using the Patient Activation Measure (PAM-13), a 13-item validated tool to assess an individual’s knowledge, skill, and confidence in managing their health [24]. Items are summed up to a total score, with higher scores indicating greater patient activation.

### Statistical analysis

Data were analysed using Stata, version 17 (StataCorp, College Station, Texas, USA). Descriptive statistics for demographics, lifestyle factors, and health outcomes of both excluded and study samples are summarised in Table 1. Comparisons between excluded and study samples at baseline were made to assess selective biases, using the T-test for continuous variables and the Chi-square test for categorical variables. To explore changes in lifestyle factors and health outcomes at the 3-year and 5-year follow-ups compared to baseline, a generalised estimating equation (GEE) model with a Gaussian family and identity link was fitted to each variable, with an exchangeable working correlation matrix to account for within-individual correlations. Standardised coefficients (β) and odds ratios (OR) were estimated for continuous and categorical variables, respectively. 95% confidence intervals were constructed around effects.

## Results

Of the 95 participants at baseline, 60 (63.2%) completed follow-up surveys at both the 3-year and 5 follow-ups (Table 1). Baseline characteristics were similar between the study sample (*N* = 60) and the excluded sample (*N* = 35), with the exception of age; participants who completed follow-ups were older than those in the excluded sample at baseline. Age was therefore included as a confounder in the analyses. Most participants were female, had a university education, were in paid employment, and were partnered. The majority had relapsing-remitting MS (RRMS).

### Changes in lifestyle factors and DMT use

Significant improvements were observed in several lifestyle risk factors from baseline to the 3-year follow-up, with many of these changes sustained or further improved at the 5-year follow-up (Tables 1 and 2). Significant increases at both follow-up points were seen in non-consumption of dairy and meat, omega 3 supplementation, and non-smoking status. Diet quality increased significantly from baseline to 3-year (β = 3.96, *p* < 0.001), although the level trended lower at 5-year (β = 2.12, *p* = 0.063). Proportions engaging with weekly meditation increased over follow-up, from 35 to 46% (OR = 1.77, *p* = 0.071) and to 48% at 5-year (OR = 1.35, *p* = 0.369). Vitamin D supplementation, DMT use, and physical activity showed no significant changes at either follow-up.

**Table 2:** Changes in lifestyle risk factors and DMT use from baseline to 3- and 5-year follow-ups (*n* = 60)

Lifestyle factors	Year	Odds Ratio	95% CI	*p*-value
DHQ diet quality score^a^	Baseline	Reference		
	3-yr follow-up	**3.96**	**(1.87**,** 6.06)**	**0.000**
	5-yr follow-up	2.12	(-0.13, 4.38)	0.063
No dairy/meat consumption	Baseline	Reference		
	3-yr follow-up	**4.10**	**(1.24**,** 6.97)**	**0.001**
	5-yr follow-up	**2.75**	**(0.86**,** 4.63)**	**0.004**
Vitamin D supplementation	Baseline	Reference		
	3-yr follow-up	1.17	(0.35, 1.98)	0.663
	5-yr follow-up	0.88	(0.25, 1.51)	0.728
Meditation at least weekly	Baseline	Reference		
	3-yr follow-up	1.77	(0.69, 2.85)	0.071
	5-yr follow-up	1.35	(0.47, 2.22)	0.369
Flaxseed oil supplementation	Baseline	Reference		
	3-yr follow-up	**7.18**	**(2.34**,** 12.03)**	**0.000**
	5-yr follow-up	**7.67**	**(2.28**,** 13.06)**	**0.000**
Moderate or high physical activity	Baseline	Reference		
	3-yr follow-up	1.39	(0.32, 2.46)	0.404
	5-yr follow-up	1.75	(0.29, 3.21)	0.189
Disease-modifying therapy use	Baseline	Reference		
	3-yr follow-up	1.04	(0.32, 1.77)	0.905
	5-yr follow-up	0.69	(0.21, 1.16)	0.290
Non-smoking	Baseline	Reference		
	3-yr follow-up	**3.80**	**(1.96**,** 5.64)**	**0.000**
	5-yr follow-up	**3.92**	**(2.02**,** 5.82)**	**0.000**

Note.^a^Mean differences and 95% confidence intervals for diet quality total score. Odds Ratios (between baseline and 3-year and baseline and 5-year) and 95% confidence intervals are presented for other lifestyle risk factors. Sample size at baseline, 3-year and 5-year follow-up = 61. Bold numbers indicate significant results, indicated by *p* < 0.05 and 95% confidence intervals that do not contain zero. Abbreviation: MSIS = Multiple Sclerosis Impact Scale; PAM = Patient Activation Measure; PDDS = Patient-determined disease steps

### Changes in health outcomes

As in Table 3, improvements in health outcomes were observed at the 3-year follow-up and were largely sustained at the 5-year follow-up: mental health QoL, physical health QoL, and self-efficacy all increased significantly from baseline to the 3-year follow-up and these improvements were sustained at the 5-year follow-up. The QoL subdomains for role limitations due to mental or physical problems were substantially improved at the 5-year timepoint compared with baseline, as were the mental subdomains of health distress and social function, and the physical subdomains of pain and health perception (data not shown). The level of physical impact of MS, as measured by MSIS-PHY, statistically reduced from baseline to 3-year, and trended at 5-year. No significant change in disability, assessed according to the PDDS, was observed at follow-up timepoints.

**Table 3:** Changes in patient-reported outcomes from baseline to 3- and 5-year (*N* = 61)

Health outcomes	Year	Mean difference	95%CI	*p*-value
Level of disability (PDDS)	Baseline	Reference		
	3-yr follow-up	-0.11	(-0.40, 0.18)	0.464
	5-yr follow-up	-0.17	(-0.51, 0.16)	0.311
Physical impact (MSIS-PHY)	Baseline	Reference		
	3-yr follow-up	**-3.71**	**(-6.63**,** -0.78)**	**0.013**
	5-yr follow-up	-2.90	(-6.14, 0.34)	0.079
Mental health composite QoL	Baseline	Reference		
	3-yr follow-up	**8.82**	**(4.02**,** 13.63)**	**0.000**
	5-yr follow-up	**6.85**	**(1.79**,** 11.89)**	**0.008**
Physical health composite QoL	Baseline	Reference		
	3-yr follow-up	**10.54**	**(6.70**,** 14.38)**	**0.000**
	5-yr follow-up	**7.31**	**(3.07**,** 11.54)**	**0.001**
Self-efficacy (PAM-13)	Baseline	Reference		
	3-yr follow-up	**2.44**	**(0.88**,** 4.01)**	**0.002**
	5-yr follow-up	**1.87**	**(0.20**,** 3.58)**	**0.029**

Note: Mean differences (between baseline and 3-year and baseline and 5-year) and 95% confidence intervals are presented. Sample size at baseline, 3-year and 5-year follow-up = 61. Bold numbers indicate significant results (*p* < 0.05). Abbreviation: MSIS-PHY = Physical impact of Multiple Sclerosis Impact Scale; PAM = Patient Activation Measure; PDDS = Patient-determined disease steps; QoL = quality of life

Sensitivity analyses were conducted with participants providing data at baseline and 3-year follow-up (*n* = 79, 83%), or baseline and 5-year follow-up (*n* = 67, 71%), with this sample who provided data at all timepoints (*n* = 60, 63%). The results (not shown) revealed that there were no material differences from the main results presented here, except for meditation and MSIS-PHY. The odds of weekly meditation increased significantly from baseline to 3-year follow-up (*n* = 79, OR = 2.01, *p* = 0.013), and MSIS-PHY showed a 3-point reduction at 5-year follow-up (*n* = 67; β=-3.08, *p* = 0.040). These outcomes were non-significant in the main results, likely due to restricted sample sizes.

## Discussion

This current study provides evidence for prospective changes associated with a brief educational workshop, demonstrating changes in risk factors and improvements in health outcomes over time, within the previously reported STOP MS study [16]. Current findings extend upon a previous 1- and 3-year follow-up of this subset of people with MS attending a residential educational lifestyle workshop on lifestyle modifications [17]. In keeping with this previous study, current findings demonstrate that the delivery of this brief intervention corresponded to improved health outcomes, sustained over the longer follow-up period to 5-years.

Sustained improvements were identified in several modifiable lifestyle risk factors: diet quality; particularly related to non-consumption of meat and dairy, omega 3 supplementation, and smoking cessation. Whilst individuals reported increased engagement with stress-reducing activities, this was non-significant. Findings align with a previous study of workshop participants within the HOLISM study, which reported that attending this intervention was associated with the adoption and maintenance of lifestyle behaviours such as omega-3 supplementation and dairy non-consumption at the 5-year follow-up [25]. Short-term effects of lifestyle interventions in enhancing healthy lifestyles have been reported in previous studies [15]. Our study adds to the evidence base by showing that adherence to lifestyle improvements can be sustained in the long-term.

This study allows for a more detailed examination of changes in patient-reported outcomes in this subset of participants. Previously reported improvements in QoL in the larger sample [16] are also evident in this subset. Importantly, participants in the current study reported clinically meaningful improvements in both mental and physical QoL, which were sustained to 5 years. This aligns with limited findings from another multimodal lifestyle intervention which reported improved QoL from baseline to 12 months in 19 people with progressive MS. In our study, the physical impact of MS was significantly reduced from baseline to 3 years, and this trend continued towards the 5-year follow-up. A multimodal intervention including a modified diet, nutritional supplements, and exercise has reported improvements in walking performance and balance over 12 months post-intervention [26]. Additionally, decreased MSIS-PHY scores were observed among participants attending multimodal inpatient rehabilitation [27]. Given that MS often leads to declining QoL and function over time [28, 29], our results showing long-term improvements are relevant and important to people with MS and clinicians alike.

The current investigation identified sustained changes in other relevant health outcomes, particularly improvements in self-efficacy at both timepoints, and stable self-reported disability over this period. Importantly, this group continued their use of DMTs, reinforcing the education provided at the workshop about the potential benefits of an integrated approach to management.

The marked increase over recent decades in research– observational and interventional – regarding the potential influence of lifestyle risk factors on MS disease activity and progression [4], strongly suggests the need for more well-designed intervention studies into this potential therapeutic avenue. Anecdotally, however, clinicians report difficulties in facilitating lifestyle change [30]. The current study suggests that facilitating sustained change is possible in the setting of an intensive live-in multimodal workshop, with facilitators experienced in this delivery and with lived experience of MS. While replicating such an intervention in other settings may not always be feasible, these findings highlight the potential value gained from a brief education intervention. Future research should explore the mechanisms through which lifestyle changes can be achieved and maintained, providing insights into the conditions and effectiveness of interventions, including brief ones, that facilitate change. For instance, it is unclear whether involving education facilitators with lived experience of MS may have an additive effect on personal learning and the likelihood that individuals adopt and maintain changes.

Separately, authors have qualitatively investigated the challenges experienced by people with MS attending these workshops [31]. Notwithstanding these challenges, qualitative findings suggest that individuals’ efforts in modifying lifestyle behaviours were positively impacted by the sense of hope and improved personal control gained through a lifestyle intervention. Further, authors have explored the experiences of partners of those people with MS attending these workshops, identifying that partners also experience significant positive psychological changes [32], improving their growing commitment to better health and wellbeing [33], more confidence in the future [34], and relationship improvements [35] that suggest wider benefits of such an intensive intervention. We have also qualitatively analysed free text data from a very large (> 1,000 participants) international sample of people with MS [36]. Despite the challenges cited by many to making significant lifestyle changes, many reported increasing hope and optimism and a sense of control stemming from engaging with lifestyle modification. These findings correspond with the improved self-efficacy observed in this study and suggest that a focus on empowerment and patient-centred care is a significant component of this multimodal intervention. Finally, based on the live-in brief intervention, a web-based educational lifestyle intervention was developed [37]. Existing qualitative and quantitative findings underline the feasibility and ease of this platform for information-seeking on lifestyle modification [38, 39], with motivators for engagement including altruism and personal health [40]. Further, findings also highlighted the psychological impact of the web-based intervention for increasing a personal sense of control over MS, greater illness coherence, and improved self-efficacy for disease management [41]. Together, current findings, contextualised by other qualitative evidence in MS, form a strong evidence-base for the feasibility and efficacy of lifestyle interventions.

### Limitations

While we report findings from an intervention study, changes in this group of participants were only compared to baseline over a time-interval, with no control group, limiting the extent to which causation can be inferred. Given the nature of the context of the intervention, a control intervention was not feasible. Despite the suggested benefits seen here, the evidence is therefore limited by the study design. Additionally, the current study is based on a small group of self-selected participants who attended an intensive live-in workshop. These participants are likely motivated to engage in lifestyle modifications and self-management of MS. The population is therefore conditioned by an important selection bias, and had a significant drop out rate. We assessed differences between the two populations at baseline but not during the 5-year period; this may have missed participants’ problems during this period. In addition, dichotomous outcomes generally have greater clinical meaning than a reduction on a continuous scale as measured in our study.

Associations between changes in lifestyle factors and patient-reported outcomes, particularly the cumulative effects of modifications on multiple lifestyle factors as shown in HOLISM [11], were not directly assessed in this study due to the limited sample size. Authors have attempted to address these limitations within a large online international randomised controlled trial (RCT) in which participants are randomly assigned to either a control (standard care) course or an intervention (lifestyle change) on a 1:1 allocation basis [37. Results from early ongoing follow-up are currently being analysed. It is relevant to note that the intervention in the current study was a single, one-off five-day event, with no follow up or reinforcement, apart from regular data collection. While this highlights the potential benefits of brief educational interventions, additional workshops or regular support may enhance commitment to lifestyle changes and improve health outcomes.

## Conclusion

Study findings show that addressing modifiable lifestyle risk factors is a feasible strategy for people with MS. Improvements in lifestyle and patient-reported outcomes subsequent to an intensive, but brief educational lifestyle intervention were sustained over time. Findings illustrate the potential value of lifestyle modification as an adjunct to conventional therapies in managing MS.
